# Type 2 Diabetes Causally Reduces Circulating Vitamin D Levels: A Multi-Ancestry Mendelian Randomization Study

**DOI:** 10.3390/nu18121944

**Published:** 2026-06-16

**Authors:** Madhusmita Rout, Piers Blackett, Dharambir K. Sanghera

**Affiliations:** 1 Department of Pediatrics, College of Medicine, University of Oklahoma Health Sciences Center, Oklahoma City, OK 73104, USA; madhusmita-rout@ou.edu (M.R.); piersblackett@att.net (P.B.); 2 Fellow of the American Academy of Pediatrics (FAAP), Department of Pediatrics, College of Medicine, University of Oklahoma Health Sciences Center, 940 Stanton L. Young Blvd., Rm 317 BMSB, Oklahoma City, OK 73104, USA; 3 Fellow of the National Lipid Association (FNLA), Department of Pediatrics, College of Medicine, University of Oklahoma Health Sciences Center, 940 Stanton L. Young Blvd., Rm 317 BMSB, Oklahoma City, OK 73104, USA; 4 Department of Pharmaceutical Sciences, University of Oklahoma Health Sciences Center, Oklahoma City, OK 73104, USA; 5 Department of Physiology, College of Medicine, University of Oklahoma Health Sciences Center, Oklahoma City, OK 73104, USA; 6 Oklahoma Center for Neuroscience, University of Oklahoma Health Sciences Center, Oklahoma City, OK 73104, USA; 7 Harold Hamm Diabetes Center, University of Oklahoma Health Sciences Center, Oklahoma City, OK 73104, USA; 8 Fellow of the American Heart Association (FAHA), Department of Pediatrics, College of Medicine, University of Oklahoma Health Sciences Center, 940 Stanton L. Young Blvd., Rm 317 BMSB, Oklahoma City, OK 73104, USA

**Keywords:** vitamin D deficiency, type 2 diabetes, cardiometabolic risk, Mendelian randomization, polygenic scores

## Abstract

**Background:** Vitamin D (25(OH)D) deficiency affects over one billion people globally and is associated with type 2 diabetes (T2D) and cardiometabolic diseases. However, causal relationships remain unclear, as vitamin D supplementation has shown limited benefit in reducing the risk of T2D. Genetic studies have identified variants that influence circulating 25(OH)D levels, but whether genetically determined vitamin D status predicts cardiometabolic outcomes remains uncertain. **Methods and Results:** Using multi-ethnic populations from the UK Biobank (471,861) and the Asian Indian Diabetic Heart Study (3486), we performed genome-wide univariate and polygenic risk score (PRS)-based bidirectional MR analyses to determine the causal association between vitamin D and T2D. A polygenic score of vitamin D–raising alleles did not affect the risk of T2D or cardiovascular disease. In contrast, a higher T2D PRS was strongly associated with an increased risk for 25(OH)D deficiency. Genetically instrumented per SD increase in T2D PRS was predicted to significantly (*p* = 9.5 × 10^−31^) reduce circulating 25(OH)D (β = −9.1 nmol/L; 95% CI: −8.9 to −9.3). The ancestry-specific univariate MR and sensitivity analyses confirmed that vitamin D levels reduced significantly with increasing T2D risk across all ancestries. **Conclusions:** Our findings suggest low circulating vitamin D levels are unlikely to causally predict T2D risk but may serve as a marker for secondary prevention in endocrine and cardiovascular health. Instead, genetic susceptibility to T2D appears to contribute to vitamin D insufficiency, which may lead to cardiovascular complications. Further studies are needed to clarify the mechanisms underlying vitamin D deficiency in diabetes.

## 1. Introduction

Vitamin D (25(OH)D) deficiency has emerged as a major global public health crisis, affecting 30–50% of the world’s population [1,2]. Vitamin D is not only important for musculoskeletal health but also plays a crucial role in various physiological functions. This includes its involvement in common cancers, cardiovascular disease (CVD), type 2 diabetes (T2D), autoimmune conditions like type 1 diabetes and multiple sclerosis, as well as Alzheimer’s and Parkinson’s diseases [3,4,5,6]. While limited sun exposure is a major cause of low vitamin D, as ultraviolet (UVB) rays are required for skin to synthesize vitamin D, ethnic and genetic differences may also affect the vitamin D status of different populations, including Chinese [7], Asian Indians [8], and African Americans [9]. A high prevalence of vitamin D deficiency has been reported among pregnant women in the Middle East, India, Pakistan, and Ethiopia [10]. People living in Indian Sub-continent require twice as much UVB exposure to produce enough vitamin D compared to Caucasians, even when the climate in India and South Asia is mostly sunny throughout the year [11]. Dietary habits such as the reduced intake of fruits, vegetables, fatty fish, egg yolks, cereals, and milk may also have contributed to vitamin D deficiency [12]. Vitamin D deficiency has been implicated in the increased risk of cardiometabolic diseases through multiple studies performed in diverse cohorts of European (EU), Australian, Asian, and American populations [8,13,14,15,16]. However, the causal association and the exact biological mechanism linking low 25(OH)D levels to cardiometabolic disease risk remain unknown [17]. Clinical trials have also shown that vitamin D supplementation does not reduce the risk of T2D [18,19] even though the environmental and genetic factors account for 50–80% of the variability in 25(OH)D levels [20,21].

Genome-wide association studies (GWASs) have identified multiple loci associated with circulating 25(OH)D levels and, interestingly, most robustly associated gene variants (identified mainly in large GWAS originating from EUs) map near genes involved in vitamin D synthesis, transport, or metabolism, such as *DHCR7/NADSYN1*, *CYP2R1*, *GC*, and *CYP24A1* [22,23,24]. In addition to the contribution of these pathway genes, studies have identified the genome-wide common variant associations and ancestry-specific associations that regulate circulating 25(OH)D, exhibiting a positive and significant association between 25(OH)D-raising alleles (as polygenic scores or PGS) and circulating 25(OH)D levels [25,26,27]. However, it is still unclear if the genetically increased 25(OH)D by genome-wide variants can predict the cardiometabolic outcomes.

Mendelian randomization (MR) studies provide a framework to assess the causal relationship between 25(OH)D levels and cardiometabolic disease outcomes, while minimizing confounding factors such as geographic location, diet, and vitamin D supplementation, and accounting for reverse causation. Several studies have identified causal links between genetically determined 25(OH)D levels and the risk of conditions including psoriasis, atopic dermatitis, vitiligo, Graves’ disease, cataracts, and esophageal cancer in EU populations [28,29]. However, studies investigating the genetic relationship between 25(OH)D levels and T2D, including potential reverse effects, have produced inconsistent results [30]. To explore whether 25(OH)D insufficiency predisposes people to T2D, we previously performed a bidirectional candidate-gene MR study and meta-analysis using three candidate gene variants associated with T2D (*IGF2BP2*, *TCF7L2*, and *KCNQ1*) and three GWAS variants from vitamin D pathway genes (*GC*, *CYP2R1*, and *DHCR7*). Our MR study found no causal evidence between T2D and 25(OH)D across eight multiethnic study cohorts comprising 59,890 individuals [31].

This study aimed to further investigate the putative role of vitamin D insufficiency in predicting endocrine and cardiometabolic health risks using genome-wide variants and polygenic score analysis, and to identify directional effects across distinct datasets. The first dataset consists of 471,861 individuals from the UK Biobank (UKBB), including 459,143 EU, 9372 South Asians (SAs), and 3346 Africans (AFs). The second population comprises a well-characterized cohort of 3486 individuals from the Asian Indian Diabetic Heart Study/Sikh Diabetes Study (AIDHS/SDS), with serum 25(OH)D levels and genome-wide genotypes available for all.

## 2. Methodology

### 2.1. Study Subjects

A total of 475,347 individuals with 25(OH)D levels from the UKBB and AIDHS/SDS were used for this study. We used genome-wide genotype and phenotype data from the UKBB recruited during the period between 2006 and 2010 in individuals of EU (*n* = 459,143), SA (*n* = 9372), and AF (*n* = 3346), following the approval (Application #78635) described previously [32,33]. Serum 25(OH)D levels were measured by chemiluminescence immunoassay (DiaSorin LIAISON XL, Saluggia, Italy) in nmol/L [34]. T2D was characterized by physician-diagnosed disease phenotype and glycated hemoglobin (HbA1c) levels. Coronary artery disease (CAD) was classified based on physician-diagnosed vascular/heart problems. Additionally, we studied 3486 subjects from the AIDHS/SDS who were available with genome-wide genotype data and serum 25(OH)D levels [8,26,31,35]. The Sikh population is a relatively homogeneous endogamous community from Northern India recruited between 2002 and 2010. Sikhs are primarily non-smokers, and ~50% of them are vegetarians. However, the incidence of cardiometabolic diseases in Sikhs and SAs has markedly increased over the past two decades [36,37]. T2D was diagnosed based on their medical records, including symptoms and use of antidiabetic medications, and in accordance with the American Diabetes Association guidelines described earlier [38,39]. Non-diabetic controls were selected based on a fasting blood glucose (FBG) < 100.8 mg/dL (5.6 mmol/L) or a 2 h glucose < 141.0 mg/dL (7.8 mmol/L) as previously described [35,39,40]. CAD was considered if there was use of nitrate medication (nitroglycerine), electrocardiographic evidence of angina pain, coronary angiographic evidence of severe (greater than 50%) stenosis, or echocardiographic evidence of myocardial infarction. The diagnosis was based on the date of coronary artery bypass graft (CABG) or angioplasty and medication usage obtained from patient records, as described previously [26,39]. Body mass index (BMI) was calculated as [weight (kg)/height (m^2^)]. Waist and hip circumferences at the abdomen and the hip, respectively, were recorded using a tape measure. For using BMI thresholds for obesity, we used the World Health Organization’s (WHO) guidelines [41]. Blood pressure (BP) was measured twice after a 5 min seated rest period with the participant’s feet flat on the floor. Serum lipids [total cholesterol (TC), triglycerides (TGs), high-density lipoprotein cholesterol (HDL-C), and low-density lipoprotein cholesterol (LDL-C)] were measured using standard enzymatic methods (Roche, Basel, Switzerland) as described previously [26,39,42,43,44]. Vitamin D levels were measured in 10 μL of serum using standard monoclonal antibody-based ELISA kits from ALPCO Diagnostics (Salem, NH, USA) across the entire AIDHS/SDS cohort, as described previously (8), in nmol/L. A standard curve was used over a range of concentrations (2-fold dilutions), and any sample that fell outside the range was repeated. All participants in this study were recruited after providing written informed consent, and the study was approved by the institutional review boards (IRBs). All AIDHS/SDS protocols and consent documents were reviewed and approved by the University of Oklahoma Health Science Center’s IRB #2911 (approved on 11.27.2021) and by the Human Subject Protection (Ethics) Committees at the participating hospitals and institutes in India, as described previously [45,46,47]. All human studies reported in this manuscript adhere to the principles of the Declaration of Helsinki (1975, revised in 2013).

### 2.2. Genotyping, Imputation, and Quality Controls

For genetic analysis, we used imputed data released by the UKBB for EU, AF, and SA subjects and excluded outliers based on heterozygosity or genotype missingness (missing rate > 0.2) and ambiguous SNPs (MAF > 0.44). Participants with inconsistent reports and genotypic inferred sex inconsistencies or withdrawn consent were removed, as explained previously [48].

For the AIDHS/SDS, samples were genotyped using the Illumina 660W Quad BeadChip, Illumina Global Screening Arrays (GSA) (Illumina, San Diego, CA, USA), and GSA with multi-disease content (GSA+) arrays as described previously [26,40,49]. Samples with genotyping call rate < 95%, cryptic relatedness, population outliers, strand-flip, departures from Hardy–Weinberg equilibrium (HWE) (*p* < 10^−7^), or MAF < 5% were excluded before imputation because rare and very rare variant imputation creates statistical noise and is technically unreliable, especially in AIDHS due to the absence of a population-specific reference panel. To increase genome coverage, data were imputed using Minimac4 [50] (https://imputationserver.sph.umich.edu/, accessed on 4 April 2024) with TOPMED r3 multiethnic reference panel in NCBI Build 38 (hg38) coordinates as reported previously [32,33]. For the AIDHS datasets, imputations were performed separately for all subsets (Batch 1 Discovery), Batch 2, and Batch 3, as these were genotyped using different Illumina chip arrays. We applied the same pre- and post-imputation QC procedures across all batches using identical coordinates (GRCh 38), strand alignment, and variant annotation, including the original genotyping chip platform, and using genomic control PCs as covariates. Approximately 3.92 million variants were common among the three batches that survived the post-imputation QC (excluding an imputation certainty info score < 0.5, MAF < 0.001, and HWE in controls (*p* < 1 × 10^−6^) and strand-ambiguous A/T or C/G SNPs at high MAF > 0.44). These variants were used to construct PGS/PRS. The genetic principal components (PCs) were estimated from our Sikh population, as the existing HapMap2, HapMap3, and 1000 Genomes data do not include data from Punjabi Sikhs, as described previously [39,49]. PCA plot was constructed for all the population (UKBB EU, SA, AF and AIDHS/SDS) using SNPRelate package in R [51]. To ensure robust clustering, outlier samples were removed if they exceeded three standard deviations from the mean across any of the top 10 principal components (Supplementary Figure S2).

### 2.3. Genome-Wide Genetic Score Construction and Analysis

Ancestry-specific PGS for 25(OH) D-raising alleles were constructed using candidate variants derived from genome-wide genotypes of UKBB and AIDHS/SDS. To construct EU-ancestry PGS, we used summary statistics from Revez et al. (2020) [52] comprising 6,098,063 variants from Sunlight Consortium. To test the associations of SNPs with circulating 25(OH)D levels, linear regression and an additive genetic model were used, with the natural-log-transformed 25(OH)D level adjusted for age, gender, BMI, 10 genetic PCs, and T2D status. We excluded INDELs, duplicate and multiallelic SNPs, and SNPs with info score < 0.80, and included SNPs with MAF > 0.01 and MAF < 0.45. After regression analysis, SNPs with *p* < 10^−4^ were chosen. After linkage disequilibrium (LD) clumping using R^2^ ≤ 0.25 and a 500 Kb distance [32,53]. A total of 2179 SNPs were used for the construction of the PGS. The EU-derived PGS performed poorly in SAs from AIDHS/SDS and the UKBB. We constructed ancestry-specific PGS using ~15 million variants from the AIDHS/SDS tested and trained on discovery (*n* = 1616) and validation (*n* = 1870) cohorts of the same Punjabi ethnicity, and 9372 SAs from UKBB. A total of 2051 SNPs were chosen for the construction of the PGS using the same selection criteria and LD clumping and regression analysis model as described for EU. For the construction of the AF PGS, we used GWAS summary statistics of 23,615,737 SNPs derived from African ancestry from Wang et al. 2023 [54] for vitamin D. The individual-level regression coefficients were multiplied by the number of risk alleles to compute the PGS as described previously [31,32,33]. The weighted PGS was calculated using the following Equation (1):

(1) $${PGS}_{j}=\sum_{i}^{N}\;\beta_{i}*\;{dosage}_{ij}$$

where *N* is the number of SNPs in the score, β_i_ is the effect size (or beta) of variant i, and dosage is the number of copies of SNP in the genotype of individual j [55]. The polygenic risk score (PRS) for T2D was constructed using the summary statistics data from O’Connor et al., [56] which was derived from 312,646 individuals of EU ancestry, using a methodology similar to that described above for all ethnic groups. For SAs, we constructed ancestry-specific T2D PRS using ~15 million variants from the AIDHS/SDS, tested in the discovery cohort (*n* = 1616) and trained on the validation cohorts (*n* = 1870) of the same Punjabi ethnicity, and 9372 SAs from UKBB. A total of 2921 SNPs were selected for PRS construction using the same selection criteria, LD clumping, and regression analysis model as described above for all ethnic groups [33]. For the construction of the AF T2D PRS, we used GWAS summary statistics for 6 million SNPs derived from AF ancestry, comprising 50,251 T2D cases and 103,909 controls, from Suzuki et al. 2024 [57].

### 2.4. Statistical Analysis

The clinical and demographic variables were summarized as means for continuous variables and percentages for categorical variables, using SPSS version 31 (IBM, New York City, NY, USA). Multivariate linear regression analyses were performed to assess the impact of 25(OH)D PGS on T2D, CAD, acute ischemic stroke (AIS), and other cardiometabolic risk factors (e.g., waist, waist-to-hip ratio (WHR), and glucose) after adjusting for covariates such as age, sex, and BMI. To evaluate the discrimination capability at the extreme tail of the genetic score, we divided the PGS into quartiles. We then compared the extreme scores in the 4th quartile with those in the 1st quartile to assess the protective effects of genetically raised 25(OH)D on T2D, CAD, and AIS. Additionally, we assessed the effects of genetically enhanced diabetes risk (T2D PRS) on 25(OH)D levels in the UKBB (EU/AF/SA) and AIDHS/SDS cohorts.

### 2.5. MR Analysis

We performed an ancestry-specific MR analysis [58] to investigate the causal effect of T2D increasing alleles, lowering the effect of vitamin D levels. The associations between the instrumental variables (gene variants) and the exposure (T2D) and the outcome (vitamin D) are estimated from different ancestries, mainly UKBB, EU, SA, AF, and AIDHS/SDS. Three basic hypotheses were considered while conducting MR: (1) genetic instrument variables (IVs) should be robustly associated with the exposure; (2) IVs should not be directly correlated to the outcome and affect the outcome merely via the exposure without any gene pleiotropy; and (3) IV should be independent of any potential confounders. The combined SNP-specific estimates were calculated using the inverse-variance weighted (IVW) method when more than 2 associated SNPs were used as IVs. The effect sizes of vitamin D were calculated per 1-SD increase in genetically predicted T2D levels. Sensitivity analyses were performed using the MR Egger method of Burgess and Thompson [59], which is based on the hypothesis that the pleiotropic effects are independently distributed from the genetic associations with the exposure. A non-zero intercept in MR Egger is meaningful, indicating that gene pleiotropy is considered to exist. MR analyses were performed using the Two-sample MR package [60] in R version 4.3.3. We ensured that the genetic instrument was strongly associated with the exposure in the target population based on regression (beta) coefficients and *p* value/F statistics accounting for the LD and allele frequency. Based on the differences in LD and MAF, the MR sensitivity analysis selected and excluded the variants from each ancestry to ensure data harmonization and reduce pleiotropy.

We also used cumulative genetic instrumental variable methods (PGS) to obtain estimates of the causal association between circulating vitamin D levels and T2D and determined the direction of causality by performing a bidirectional MR study [31,40]. The associations between the exposure (T2D) and the outcome (25(OH)D) levels, and vice versa, are estimated from different cohorts, mainly UKBB (EU, SA, and AF) and AIDHS/SDS. The combined estimates were calculated using the conventional MR method [58,61]. In sensitivity analyses, we used the two-stage least squares (2SLSs) method to validate the causal effect and the strength of the association since the allelic score methods were used for the MR [61,62]. In stage 1, the exposure of interest is regressed on the polygenic score (controlling for covariates of age, gender, BMI, and ancestry) to obtain predicted values of the exposure. Stage 2 estimates the causal effect by regressing the predicted values of the exposure obtained from the first stage [63] and F values > 10 were considered to confirm the causal effect. All analyses were performed using PLINK 2.0 [64], SVS version 8.9.1 (Golden Helix, Bozeman, MT, USA), and SPSS version 31 (IBM, New York City, NY, USA), and R (version 4.3.3).

## 3. Results

The clinical and demographic characteristics of the UKBB and AIDHS/SDS study participants are presented in Table 1. The AIDHS/SDS individuals showed higher levels of most clinical risk traits than the UKBB cohort. For instance, the mean WHR was significantly higher for AIDHS/SDS (0.94 ± 0.08) compared to UKBB EU (0.87 ± 0.09) (*p* = 2.5 × 10^−225^). Similarly, the average blood glucose levels were significantly higher in AIDHS/SDS (134.78 + 63.72) than in UKBBEU (80.30 ± 36.89; *p* = 4.65 × 10^−325^) and UKBBSA (97.66 ± 33.94; *p* = 2.07 × 10^−196^). Similarly, triglycerides were significantly higher in AIDHS/SDS (169.87 ± 112.60) compared with UKBB (EU and AF) individuals (Table 1). The participant flow chart and study workflow is presented in Supplementary Figure S1.

**Figure 1:**
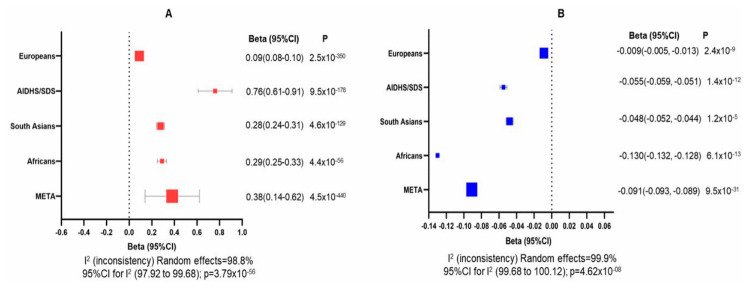
Random-effect meta-analysis showing effect sizes and confidence intervals of (**A**) Vitamin D PGS effect on vitamin D levels. Allele score regression using two-stage least squares (2-SLSs) showed a mean F = 5563.31; *p* = 3.62 × 10^−338^; % covariance = 1.172. (**B**) T2D PRS effect on vitamin D levels in UK Biobank and AIDHS/SDS cohorts. Allele score regression showed a mean F = 228.02; *p* = 1.66 × 10^−51^; % covariance = 0.049. AIDHS/SDS: Asian Indian Diabetic Heart Study/Sikh Diabetes Study; PGS: Polygenic score; PRS: Polygenic risk scores; T2D: Type 2 diabetes.

**Figure 2:**
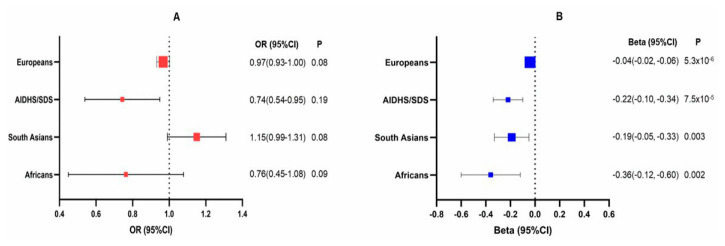
Forest plots showing effect sizes and confidence intervals of individuals with extreme PGS in the 4th quartile compared with those in the 1st quartile to determine the risk for (**A**) Vitamin D PGS effect on T2D (**B**) T2D PRS effect on vitamin D levels in UK Biobank and AIDHS/SDS cohorts.

The assessment of cumulative effects of vitamin D-raising alleles as PGS for each ancestry showed a stronger association with 25(OH)D levels, as indicated by larger effect sizes. As shown in Figure 1A the AIDHS/SDS showed the strongest allelic effect for increasing 25(OH)D levels (β = 0.76 (95% CI 0.61–0.91; *p* = 9.5 × 10^−178^), and a similar trend was observed in AF β = 0.29 (95% CI 0.25–0.33; *p* = 4.4 × 10^−56^), SA β = 0.28 (95% CI 0.24–0.31; *p* = 4.6 × 10^−129^), and EU β = 0.09 (95% CI 0.08–0.10; *p* = 2.5 × 10^−350^) (Figure 1A). Combining all cohorts, the overall meta-analysis showed a strong effect of vitamin D PGS on increasing 25(OH)D levels, β = 0.38 (95% CI 0.14–0.62; *p* = 4.5 × 10^−440^) (Figure 1A). However, the reverse effects of vitamin D-raising alleles (25(OH)D PGS) on reducing the risk for T2D were not observed in these cohorts (Supplementary Table S2b). To further evaluate if genetically increased vitamin D lowers T2D susceptibility, we divided the vitamin D-PGS into quartiles. When comparing the individuals in the 4th quartile (high 25(OH)D levels) vs. the 1st quartile (25(OH)D deficiency group), there was a marginal, non-significant decrease in the risk for T2D in the EU. Similarly, a non-significant association was observed in AIDHS/SDS and in AF (Figure 2A). Similarly, comparing the extreme quartiles of vitamin D PGS, the individuals with genetically enhanced 25(OH)D levels reduced the risk of CAD with marginal significance in EU (OR 0.96 (95%CI 0.92–1.00; *p* = 0.05). A similar but non-significant trend was observed in AIDHS/SDS and AF, indicating a decreased CAD risk with increasing 25(OH)D (Supplementary Table S1).

Conversely, the cumulative PRS score for T2D exhibited significantly lowered 25(OH)D levels across all ethnic groups. People with an increased T2D PRS had consistently lower 25(OH)D levels when compared with those with lower T2D PRS (β = −0.055; 95% CI, −0.059, −0.051; *p* = 1.4 × 10^−12^) in AIDHS/SDS and UKBB cohort EU (β = −0.009; 95% CI, −0.005, −0.013; *p* = 2.4 × 10^−9^), SA (β = −0.048; 95% CI −0.052, −0.044; *p* = 1.2 × 10^−5^), and AF (β = −0.130; 95% CI −0.132, −0.128; *p* = 6.1 × 10^−13^), respectively (Figure 1B). The combined meta-analysis of all cohorts showed significantly reduced 25(OH)D levels (β = −0.091; 95% CI −0.093, −0.089; *p* = 9.5 × 10^−31^) with increased T2D PRS (Figure 1B).

Next, we compared the extreme 4th quartile (high T2D risk) vs. the 1st quartile (low T2D risk) PRS. In the EU, 25(OH)D levels were significantly decreased (β = −0.04; 95% CI = −0.02, −0.06; *p* = 5.3 × 10^−6^) in individuals with high T2D PRS. In AIDHS/SDS, 25(OH)D levels were also significantly reduced (β = −0.22; 95% CI = −0.10, −0.34; *p* = 7.5 × 10^−5^). Similarly, SA and AF showed a significantly decreased 25(OH)D levels in the group with the extremely high T2D PRS (β −0.19; 95% CI −0.05, −0.33; *p* = 0.003) and (β −0.36; 95% CI −0.12, −0.60; *p* = 0.002), respectively (Figure 2B).

We further studied the association between ancestry-derived vitamin D PGS effects and cardiometabolic traits, revealing a significant negative association with WHR in UKBB EU (β ± SE = −0.008 ± 0.001; *p* = 8.9 × 10^−11^). Similarly, individuals with increased vitamin D PGS had a significant reduction in waist circumference in SA (β ± SE = −0.009 ± 0.001; *p* = 1.5 × 10^−13^) (Supplementary Table S2a) and also had significantly lowered glucose levels (β ± SE = −0.12 ± 0.06; *p* = 0.03) (Supplementary Table S2a) and T2D risk (OR 0.91 (95% CI 0.85–0.98; *p* = 0.009) in the AF (Supplementary Table S2b).

**Figure 3:**
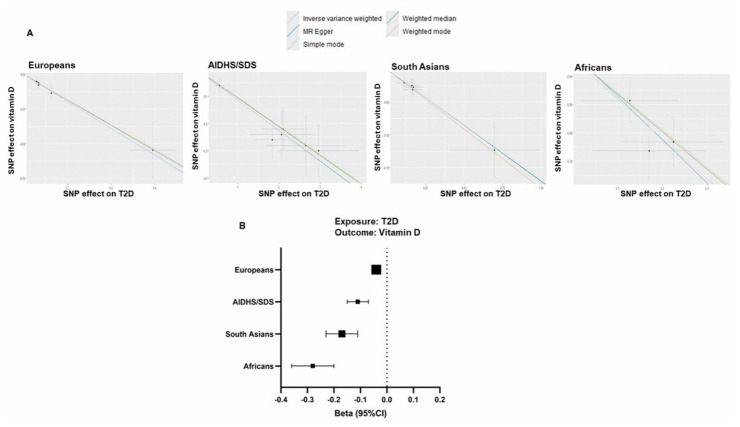
Scatter plots and forest plots showing genetic association between T2D, and vitamin D. Using univariate MR and sensitivity analysis applying the IVW, weighted median, weighted mode, maximum likelihood, and MR-Egger methods for fixed effects (FEs) and random effects (REs) (**A**) Scatter plots on top show the effect of T2D-risk SNPs on the exposure on the *x*-axis and outcome (vitamin D) on the *y*-axis with each dot represent individual SNP in UK Biobank and AIDHS/SDS cohorts. (**B**) Forest plot shows the IVW ORs and *p*-values of T2D-associated SNPs on vitamin D in UK Biobank and AIDHS/SDS cohorts.

To further evaluate the causal link between T2D and vitamin D levels, we used genetic instruments in ancestry-specific MR [61]. Using ancestry-specific univariate MR and sensitivity analysis applying the IVW, weighted median, weighted mode, maximum likelihood, and MR-Egger methods for fixed effects (FE) and random effects (RE), we tested the associations of all significantly associated T2D SNPs effect on vitamin D levels in each ancestry. We followed a strict quality-control and systematic approach to ensure that a genetic instrument derived from one ancestry was valid and strongly associated with the exposure in the target population, based on regression (beta) coefficients and *p*-values/F-statistics, accounting for LD and allele frequencies. Genetically instrumented per 1-SD increment in T2D risk was associated with a significant decrease in vitamin D levels across all cohorts. EUs from UKBB showed a significant decrease in vitamin D levels (β = −0.04; 95% CI −0.05, −0.03; *p* = 4.2 × 10^−9^). Similarly, both the SA cohorts showed a significant lowering of vitamin D levels with increasing T2D risk, in AIDHS/SDS (β = −0.11; 95% CI −0.15, −0.07; *p* = 6.5 × 10^−12^) and SAs from UKBB (β = −0.17; 95% CI −0.23, −0.11; *p* = 5.7 × 10^−8^). AFs from UKBB also showed a similar pattern with (β = −0.28; 95% CI −0.36, −0.20; *p* = 3.5 × 10^−14^) (Figure 3). The genes encoding IVs used for MR for each population are mentioned in Supplementary Table S3. Using leave-one-out analyses, we systematically removed variants to reduce heterogeneity and control for horizontal pleiotropy, and the final heterogeneity estimates after outlier removal are tabulated in Supplementary Table S4.

We also analyzed individual variants from bona fide/established candidate genes involved in vitamin D synthesis, transport, and metabolism, such as *GC*, *DHCR7*, *CYP24A1*, *CYP2R1*, *LIPC*, *CETP*, *HAL*, and *CRX*, that also showed GWAS-level significance for 25(OH)D levels in this study. We found that individuals with and without vitamin D supplements had a similar association between these variants and vitamin D levels (Supplementary Table S5a). However, no significant association of these variants was observed with other cardiometabolic risk factors in the UKBB EU (Supplementary Tables S5b–f).

Next, we constructed PGS of these candidate genes (*GC*, *DHCR7*, *CYP24A1*, *CYP2R1*, *LIPC*, *CETP*, *HAL*, and *CRX*). As expected, the targeted genetic score of candidate genes using GWAS-level significant variants revealed strong association for increasing 25(OH)D levels in each ethnicity (Supplementary Table S6a). However, the targeted vitamin D-raising PGS was ineffective in predicting protection against T2D or CAD risk (Supplementary Table S6b).

We further investigated the relationship between individual SNPs (T2D risk alleles) within the T2D PRS and their effects on vitamin D levels. We found that some of these T2D risk gene variants were associated with reduced 25(OH)D levels; however, the significance of these associations was marginal or not significant across all cohorts (Supplementary Table S7).

We also observed that circulating 25(OH)D levels did differ by T2D onset in UKBB EU, decreasing significantly from (41.38 ± 23.83) nmol/L at 17–30 years to (38.51 ± 22.05) nmol/L at 31–50 years and then increased from (41.12 ± 22.75) nmol/L at 51–60 years to (44.63 ± 22.63) nmol/L at 61–70 years (F = 79.99; *p* = 1.79 × 10^−51^). But the 25(OH)D levels did not differ for the duration of T2D in UKBB EU (F = 0.72; *p* = 0.54) (Supplementary Table S8a). In AIDHS/SDS the average 25(OH)D levels showed a significant linear increase from (34.07 ± 39.12) nmol/L to (45.21 ± 45.43) nmol/L for age of T2D onset at 13 years to 92 years (F = 6.68; *p* = 1.75 × 10^−04^), while vitamin D levels decreased significantly from (44.26 ± 45.18) nmol/L at the fresh T2D onset (0 year duration) to (28.66 ± 33.03) nmol/L with >11 years of T2D duration (F = 15.54; *p* = 5.58 × 10^−10^) (Supplementary Table S8b).

Additionally, as expected, the T2D PRS was associated with an increased risk of CAD across all cohorts. UKBB EU (β ± SE = 0.018 ± 0.003; *p* = 4.9 × 10^−10^), AIDHS/SDS (β ± SE = 10.773 ± 3.066; *p* = 4.4 × 10^−04^), UKBB SA (β ± SE = 0.238 ± 0.046; *p* = 1.7 × 10^−07^), and UKBB AF (β ± SE = 0.955 ± 0.325; *p* = 0.003) (Supplementary Table S9) [65]. Likewise, the T2D PRS was linked to a significantly increased risk of stroke across all UK Biobank cohorts: EU (β ± SE = 0.091 ± 0.002; *p* = 3.1 × 10^−308^), SA (β ± SE = 1.178 ± 0.050; *p* = 4.9 × 10^−118^), and AF (β ± SE = 4.503 ± 0.211; *p* = 1.2 × 10^−94^). Although the effect of T2D PRS on AIS was in the same direction, it was not significant in AIDHS/SDS (β ± SE = 1.349 ± 0.795; *p* = 0.84), perhaps because of the small number of stroke cases (*n* = 111) in this cohort. (Supplementary Table S9).

## 4. Discussion

This study aimed to determine the critical role of vitamin D insufficiency in predicting the risk of cardiometabolic diseases, utilizing diverse ethnic cohorts and advanced genome-wide approaches. Using candidate gene variants and cumulative genome-wide polygenic scores, our findings did not confirm whether vitamin D insufficiency predisposes people to developing T2D and other cardiovascular complications; rather, these data suggest that vitamin D may serve only as a marker for secondary prevention in endocrine and cardiometabolic health. Although ancestry-derived 25(OH)D PGS were strongly associated with circulating 25(OH)D levels in this multi-ethnic study, they did not translate into predictive value for significant protection against cardiometabolic risk. This pattern was consistent across multiple analytical frameworks, including continuous and quartile-based analysis. On the other hand, our MR findings (univariate MR and PGS-derived MR) suggest that people with T2D would be more likely to develop vitamin D deficiency and subsequent cardiovascular complications.

Vitamin D deficiency is an established risk factor for T2D, CAD, and other cardiometabolic diseases [66,67]. We earlier reported a very high prevalence of vitamin D deficiency in this Punjabi diabetic cohort (AIDHS/SDS), showing a strong association of reduced 25(OH)D with T2D and other cardiometabolic risk traits [8,26,68]. In a bidirectional MR study conducted by our group, using GWAS variants from three T2D candidate genes (*IGF2BP2*, *TCF7L2*, *KCNQ1*) and three vitamin D pathway genes (*GC*, *CYP2R1*, *DHCR7*) across 59,890 individuals from EU and Asian Indian ethnic groups, no causal link between T2D and 25(OH)D was found [31]. Our current findings again failed to establish a causal relationship between a genetically instrumented increase in 25(OH)D and reduced susceptibility to T2D and other cardiovascular diseases and traits, after including genome-wide polygenic scores and an expanded study cohort of more than 450,000 individuals from UKBB. These results support the prior published studies from other groups, where MR analyses have shown no causal association between vitamin D levels and the risk of T2D [69], including MR analysis of large consortia studies (European Prospective Investigation into Cancer and Nutrition [EPIC]–InterAct, EPIC-Norfolk, EPIC-CVD, Ely, and the SUNLIGHT consortium) [70]. Similarly, a study by La Barrera et al. (2023) found that increased 25(OH)D levels did not affect the risk of youth-onset T2D [71]. Even when restricting the PGS analysis to variants in established vitamin D pathway genes and lipid metabolism genes, which were strongly associated with 25(OH)D levels, we observed no association with T2D and cardiometabolic traits.

On the contrary, our results demonstrate a strong causal association between genetically enhanced T2D susceptibility and significantly reduced 25(OH)D levels across multiple ethnic groups, as indicated by global univariate analysis and PRS-driven MR. A genetically instrumented per SD increase in T2D PRS would reduce 25(OH)D levels to 9.1 nmol/L with 95%CI from 8.9 nmol/L to 9.3 nmol/L (*p* = 9.5 × 10^−31^). These results were consistent with those obtained by univariate MR and sensitivity analyses using the IVW, weighted median, weighted mode, maximum likelihood, and MR-Egger methods (Figure 3). This genetic evidence also supports epidemiological findings, including studies from our group, which have consistently shown that vitamin D deficiency is more prevalent in individuals with T2D than in those without T2D across multiple populations [72,73]. Prospective studies have shown that vitamin D deficiency accelerates the development, progression, and severity of T2D [66]. In patients with T2D, circulating 25(OH)D levels are often reduced due to sequestration of vitamin D in adipose tissue, metabolic dysfunction, and diabetes-related complications [74]; however, the exact molecular mechanism remains unexplored.

Gene expression studies have reported downregulation of the vitamin D receptor (*VDR*) in individuals with diabetes, which may also contribute to reduced circulating 25(OH)D levels in T2D [75]. Hypermethylation of *VDR* and vitamin D-metabolizing genes, such as *CYP27B1* and *CYP2R1*, has been shown to reduce the expression of these genes, leading to decreased synthesis of active vitamin D, impaired calcium homeostasis, and metabolic dysfunction [76,77,78]. Similarly, reduced 25(OH)D levels have been reported in obese Saudi women due to hypermethylation of *CYP2R1* and *CYP27B1* [79]. Downregulation of the *Lrp2-Cubn-Dab2* complex disrupts renal reabsorption of vitamin D bound to the D-binding protein, which leads to increased urinary elimination of vitamin D in diabetes [80] as shown in Supplementary Figure S3. Possibly, increasing the expression of *Lrp2-Cubn-Dab2* receptors may prevent the loss of vitamin D bound to the D-binding protein through urinary elimination. Collectively, these findings underscore the need for further investigation into the biological and molecular mechanisms contributing to reduced vitamin D levels in T2D.

This study has several notable strengths. First, it investigates the impact of genome-wide vitamin D PGS on T2D and cardiometabolic disease risk across multi-ethnic populations. To our knowledge, no studies have yet explored this potentially causal connection using a genome-wide bidirectional MR approach. The genome-wide risk score information was obtained from summary statistics of large consortium studies to reduce genetic score inflation, and multiple sensitivity analyses were performed to assess the reliability of causal inference. The inclusion of diverse ethnic groups improved the generalizability of our findings and reinforced the evidence of no association between vitamin D and protection against T2D.

Similarly, this study has limitations, including a lack of diversity in the sample; over 80% of participants are from the EU. Also, the lack of genome-wide data and vitamin D measures in other diverse non-EU cohorts might have contributed to the creation of weak genetic instruments. There was observable phenotypic and genotypic heterogeneity between SA populations from UKBB and AIDHS/SDS. The SA cohort from UKBB is a random collection of SAs living in the UK, originating from all over the world, whereas the AIDHS/SDS included a homogeneous population of Punjabis, predominantly Sikhs, from North India. SAs from UKBB are extremely heterogeneous; hence, the sample composition may have limited PGS/PRS transferability. Moreover, given the modest size of the African cohort (*n* = 3346) in the UKBB and the extensive heterogeneity within the population, there was insufficient power to conduct the MR sensitivity analysis adequately. Also, both the UKBB and AIDHS/SDS participants were recruited around two decades ago, between 2006 and 2010 for the UKBB and 2002 and 2010 for the AIDHS/SDS cohorts, respectively. Vitamin D levels measured at different time points may have contributed to the variation among individuals and cohorts. Despite these limitations, our polygenic score models demonstrated robustness for predicting T2D and were significantly effective for predicting 25(OH)D status across all ethnic cohorts. Univariate MR analysis has reduced the potential bias from pleiotropy induced by polygenic instruments. At the same time, due to heterogeneity, the MR sensitivity analysis excluded many common genetic variants that contributed to the causal effect during the leave-one-out analysis, leading to a different set of variants showing the causal effect of T2D on vitamin D insufficiency across differentcohort in the univariate MR approach. Despite these limitations, validation of the PGS-driven MR results in the more sensitive univariate MR, using multiple sensitivity models, has revealed important insights into the potential causal link between T2D and vitamin D insufficiency. Validation of these findings in larger, independent cohorts from minority communities would be needed to identify putative mechanisms underlying vitamin D insufficiency in T2D.

## 5. Conclusions

In summary, our findings provide no evidence for an association between genetically increased vitamin D levels or exogenous supplementation and protection against T2D and cardiovascular risk. In contrast, our new findings from genome-wide analysis and cumulative genetic scores demonstrated that people with genetically enhanced T2D risk are more prone to vitamin D insufficiency, which may help explain T2D-related complications across ethnic groups. These findings may partly explain why individuals with T2D are 2 to 4 times more likely to develop fatal CAD, heart failure, and AIS than those without T2D. And that improved vitamin D status can be a modifier of endocrine and cardiovascular health. Given the rising prevalence of T2D, these results are timely and important, highlighting the need to deepen understanding of the biological mechanisms linking diabetes to vitamin D deficiency and the consequent cardiovascular complications that affect aging and health span.

## Figures and Tables

**Table 1:** Clinical characteristics of the UKBB and AIDHS/SDS individuals.

Trait	Europeans (*N* = 459,143)	AIDHS/SDS (*N* = 3486)	South Asians (*N* = 9372)	Africans (*N* = 3346)
Males (%)	46	55	54	51
Age (years)	56.77 ± 8.03	51.98 ± 13.27 *	53.30 ± 8.45 ^	51.00 ± 7.94
BMI (kg/m^2^)	27.40 ± 4.77	26.56 ± 4.79 *	27.16 ± 4.40 ^	29.68 ± 5.14
Waist (cm)	90.26 ± 13.51	92.23 ± 11.94 *	91.45 ± 11.86 ^	94.16 ± 11.63
Waist-to-hip ratio	0.87 ± 0.09	0.94 ± 0.08 *	0.90 ± 0.09 ^	0.88 ± 0.08
Systolic BP (mmHg)	137.98 ± 18.64	137.07 ± 28.83	129.90 ± 29.73 ^	138.66 ± 18.87
Diastolic BP (mmHg)	82.18 ± 10.12	82.51 ± 12.43	79.45 ± 17.47 ^	84.92 ± 10.83
Blood glucose (mg/dL)	80.30 ± 36.89	134.78 ± 63.72 *	97.66 ± 33.94 ^	91.99 ± 27.23
Triglycerides (mg/dL)	147.89 ± 94.52	169.87 ± 112.60 *	173.98 ± 103.35	107.29 ± 67.55
HDL-C (mg/dL)	49.03 ± 23.28	40.53 ± 14.80 *	48.79 ± 12.48 ^	53.84 ± 13.84
LDL-C (mg/dL)	131.31 ± 44.17	112.79 ± 39.04 *	129.34 ± 32.96 ^	123.96 ± 32.19
Total Cholesterol (mg/dL)	212.05 ± 61.18	184.06 ± 61.74 *	205.38 ± 43.62 ^	198.73 ± 42.34
Vitamin D levels (nmol/L)	41.23 ± 26.13	35.05 ± 26.69 *	21.36 ± 17.81 ^	27.67 ± 19.04
T2D (*N*) (%)	(22,487) (5%)	(1779) (51%) *	(1943) (21%) ^	(366) (11%)
CAD (%)	(24,887) (5%)	(663) (19%) *	(2941) (31%) ^	(204) (6%)

Values are displayed in mean ± SD; * Comparison between UKBB Europeans and AIDHS/SDS (*p* < 0.001); ^ Comparison between UKBB South Asians and AIDHS/SDS (*p* < 0.001); AIDHS/SDS: Asian Indian Diabetic Heart Study/Sikh Diabetes Study; BMI: Body mass index; BP: Blood pressure; CAD: Coronary artery disease; HDL-C: High-density lipoproteins-Cholesterol; LDL-C: Low-density lipoproteins-Cholesterol; T2D: Type 2 diabetes; UKBB: UK Biobank; Reference range for healthy individuals are as follows: BMI (Kg/m^2^): 18.5–24.9; Waist (cm): Women < 80 and Men < 94; Systolic BP (mmHg): <120; Diastolic BP (mmHg): <80; Blood glucose (mg/dL): Fasting < 100 and Random < 140; Triglycerides (mg/dL): <150; HDL-C (mg/dL): Men > 40, Women > 50; LDL-C (mg/dL): <100; Total Cholesterol (mg/dL): <200; Vitamin D levels (nmol/L): 50–125.

## Data Availability

The genome-wide genotype data associated with the Punjabi Sikh discovery, along with phenotype data, can be found at https://urldefense.com/v3/__https://www.ncbi.nlm.nih.gov/gap/advanced_search/?TERM=sanghera__;!!GNU8KkXDZlD12Q!80VGcpVpziE84NcBfNA6_5uWAIfuQrJ7qN4q1Udjs6JfGGgxcv5ZoucQj9lRsWZxv7Hjb8NIdVIQzeUVgaeobQKbxQ$[ncbi[.]nlm[.]nih[.]gov]). This data is only available under authorized access and requires an application.

## Supplementary Materials

The following supporting information can be downloaded at: https://www.mdpi.com/article/10.3390/nu18121944/s1, Figure S1: Summary of the workflow detailing the study design and the outcomes. Figure S2: (A) Principal component analysis of the AIDHS/SDS GWAS population and HapMap3 founder populations GIH, CEU, CHB, JPT and YRI. Eigenvectors demonstrate the proximity of the AIDHS/SDS GWAS population to the GIH and CEU populations, and the close matching of T2D cases and controls. (Image courtesy: Saxena R et al. Diabetes. 2013 May;62(5):1746-55) [49]. (B) Principal component analysis of the UKBB populations. The principal components: PC1 and PC2 show separate clustering of Europeans, South Asians and African populations with South Asians clustering closer to Europeans. Figure S3: Possible mechanism of vitamin D insufficiency in diabetes, renal metabolic dysfunction impairs Lrp2/Cubn/*Dab2*-mediated 25(OH)D_3_ uptake by downregulation of Cyp27b1 expression, which impairs the hydrolysis of 25(OH)D_3_ to 1,25(OH)_2_D_3_ levels and reduces the vitamin D receptor (Vdr) activation. Reduced Vdr activity impairs the vitamin D reabsorption by the kidney and increases its loss in urine. Table S1: The effect of vitamin D polygenic score (extreme 4th quartiles vs. 1st quartile) on cardiometabolic diseases in UK Biobank and AIDHS/SDS cohorts. Table S2: (a): The effect of vitamin D polygenic score on cardiometabolic traits in UK Biobank and AIDHS/SDS cohorts; (b): The effect of vitamin D polygenic score on cardiometabolic diseases in UK Biobank and AIDHS/SDS cohorts. Table S3: (a): The T2D risk SNPs used for univariate two-sample MR in Europeans; (b): The T2D risk SNPs used for univariate two-sample MR in AIDHS/SDS; (c): The T2D risk SNPs used for univariate two-sample MR in South Asians; (d): The T2D risk SNPs used for univariate two-sample MR in Africans. Table S4: Horizontal pleiotropy and Cochrane Q-statistics of the effect of T2D risk SNPs on vitamin D levels in univariate two-sample MR. Table S5: (a): The association of variants from top vitamin D genes with vitamin D levels in UK Biobank Europeans; (b): The association of variants from top vitamin D genes with waist in UK Biobank Europeans; (c): The association of variants from top vitamin D genes with waist-to-hip ratio in UK Biobank Europeans; (d): The association of variants from top vitamin D genes with Coronary artery disease in UK Biobank Europeans; (e): The association of variants from top vitamin D genes with glucose in UK Biobank Europeans; (f): The association of variants from top vitamin D genes with Type 2 diabetes in UK Biobank Europeans. Table S6: (a): The effect of vitamin D polygenic score (PGS) derived from variants representing top vitamin D associated genes on cardiometabolic traits in UK Biobank and AIDHS/SDS cohorts; (b): The effect of vitamin D polygenic score (PGS) derived from variants representing top vitamin D associated genes on cardiometabolic diseases in UK Biobank and AIDHS/SDS cohorts. Table S7: The association of T2D risk alleles with vitamin D levels in UK Biobank and AIDHS/SDS cohorts. Table S8: (a): The mean vitamin D levels across the age of T2D onset and duration of T2D in the UK Biobank Europeans; (b): The mean vitamin D levels across the age of T2D onset and duration of T2D in the AIDHS/SDS cohort. Table S9: The effect of T2D polygenic risk score on cardiometabolic diseases in UK Biobank and AIDHS/SDS cohorts.

## Abbreviations

AIS	Acute Ischemic Stroke
AIDHS/SDS	Asian Indian Diabetic Heart Study/Sikh Diabetes Study
AF	Africans
BG	Blood glucose
BMI	Body mass index
BP	Blood pressure
CABG	Coronary artery bypass graft
CAD	Coronary artery disease
CVD	Cardiovascular disease
DBP	Diastolic blood pressure
EU	European
GSA	Global Screening Arrays
GWAS	Genome-wide association studies
HDL-C	High-density lipoprotein cholesterol
HWE	Hardy-Weinberg equilibrium
LD	Linkage disequilibrium
LDL-C	Low-density lipoprotein cholesterol
MAF	Minor allele frequency
MR	Mendelian randomization
PC	Principal components
PGS	Polygenic score
PRS	Polygenic risk score
SA	South Asians
SBP	Systolic blood pressure
T2D	Type 2 diabetes
TC	Total cholesterol
TG	Triglycerides
UKBB	UK Biobank
Vitamin D	25(OH)D
WHR	Waist-to-hip ratio
