# Supplementary material for: Type 2 Diabetes Causally Reduces Circulating Vitamin D Levels: A Multi-Ancestry Mendelian Randomization Study
Source: Nutrients. 2026 Jun 16;18(12):1944. doi: 10.3390/nu18121944 (PMC13305945; doi:10.3390/nu18121944)
Supplement: Supplementary file 1 [file nutrients-18-01944-s001.zip › Supplementary Figures.pdf]

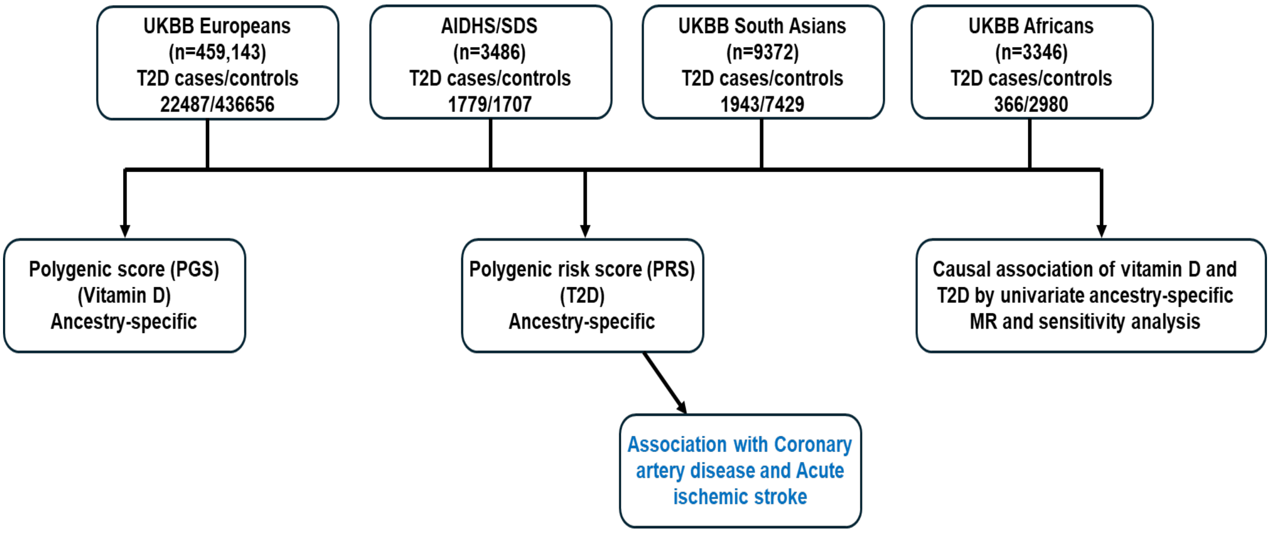

Supplementary Figure S1: Summary of the workflow detailing the study design and the outcomes.

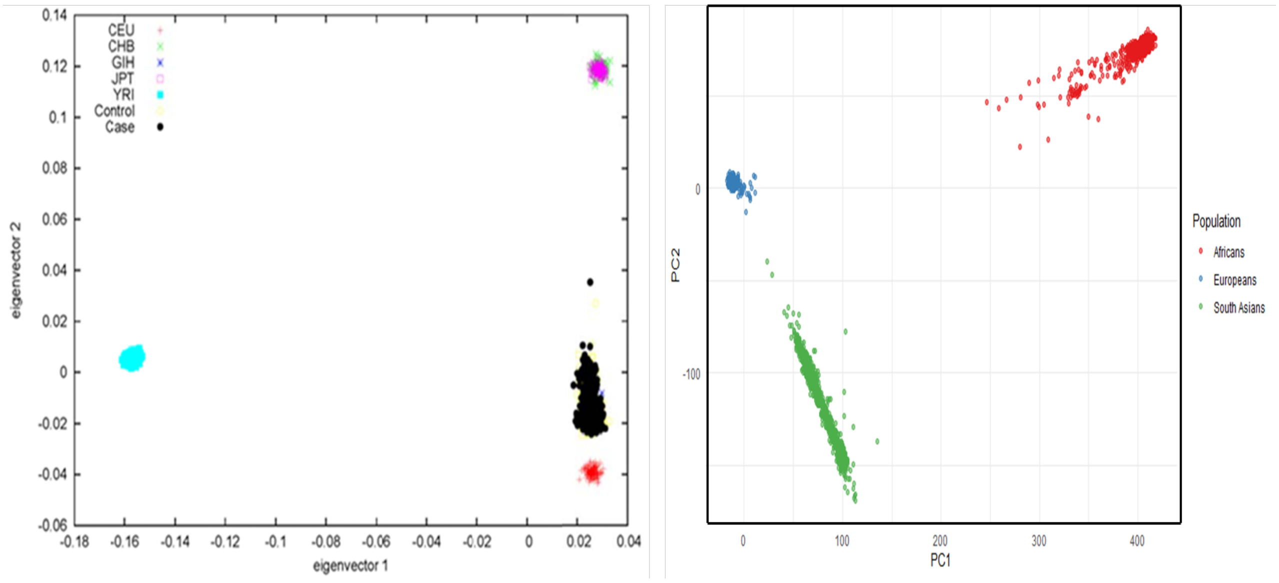

Supplementary Figure S2: (**A**) Principal component analysis of the AIDHS/SDS GWAS population and HapMap3 founder populations GIH, CEU, CHB, JPT and YRI. Eigenvectors demonstrate the proximity of the AIDHS/SDS GWAS population to the GIH and CEU populations, and the close matching of T2D cases and controls. (Image courtesy: **Saxena R et al. Diabetes. 2013 May;62(5):1746-55**) [49]. (**B**) Principal component analysis of the UKBB populations. The principal components: PC1 and PC2 show separate clustering of Europeans, South Asians and African populations with South Asians clustering closer to Europeans.

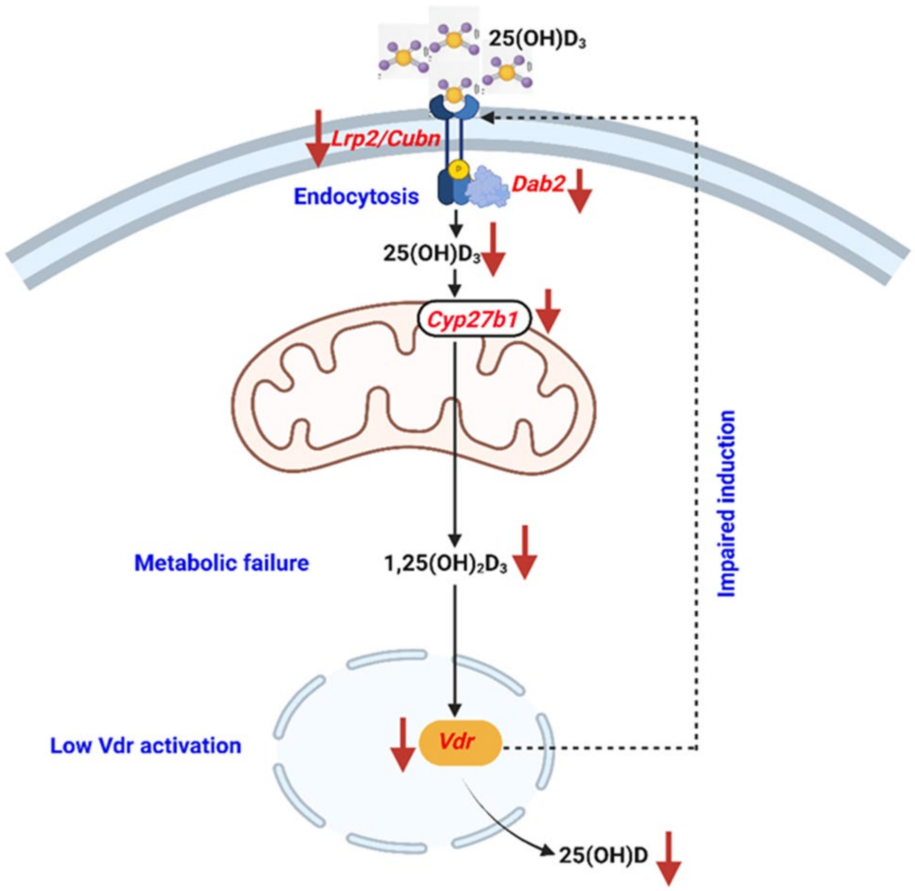

Supplementary Figure S3: Possible mechanism of vitamin D insufficiency in diabetes, renal metabolic dysfunction impairs Lrp2/Cubn/*Dab2*-mediated 25(OH)D_3 uptake by downregulation of Cyp27b1 expression, which impairs the hydrolysis of 25(OH)D_3 to 1,25$(OH)_2D_3$ levels and reduces the vitamin D receptor (Vdr) activation. Reduced Vdr activity impairs the vitamin D reabsorption by the kidney and increases its loss in urine.
